# Effects of online frame-of-reference training on assessment accuracy in the objective structured clinical examination for physical therapy students

**DOI:** 10.20407/fmj.2022-032

**Published:** 2023-08-28

**Authors:** Tetsuro Watari, Kei Ohtsuka, Yukari Suzuki, Fumihiro Matsuda, Soichiro Koyama, Naoki Aizu, Yoshikiyo Kanada, Hiroaki Sakurai

**Affiliations:** Faculty of Rehabilitation, School of Health Sciences, Fujita Health University, Toyoake, Aichi, Japan

**Keywords:** Objective structured clinical examination, Rater, Frame-of-reference training, Accuracy, Online

## Abstract

**Objectives:**

This study investigates how online frame-of-reference (FOR) training of raters of the objective structured clinical examination (OSCE) for physical therapy students affects assessment accuracy.

**Methods:**

The research was conducted in a 1-month-long randomized controlled trial.

**Participants:**

The participants were 36 physical therapists without experience assessing clinical skills using the OSCE. The training group completed the FOR training online, which was conducted once a week in two 90-minute sessions. The control group self-studied the rubric rating chart used in the assessment. As a measure of accuracy, weighted kappa coefficients were used to check the agreement between correct score and those assessment by the participant in the OSCE.

**Results:**

The scores of the training group were higher than those of the control group in both post- and follow-up assessments, showing significant differences. No significant difference was found based on the assessment time and group for the high-agreement groups. Furthermore, scores of the low-accuracy training group were higher in the post- and follow-up assessments than those in the pre-assessment, showing significant differences.

**Conclusions:**

Online FOR training of the raters of the OSCE for physical therapists improved the assessment accuracy of the raters who had low accuracy in the pre-assessment; this improvement was maintained.

## Introduction

The objective structured clinical examination (OSCE),^1^ developed in 1975 by Harden et al., is a tool that assesses the clinical skills of healthcare professionals. These professionals include nurses, physical therapists, occupational therapists, radiologic technologists, and pharmacists.^2^ The OSCE has been widely employed in healthcare professional’s student education.^3–7^ Many studies have examined its inter-rater reliability and accuracy. For example, Cohen et al. investigated the OSCE assessing the clinical abilities of surgical interns, reporting a high agreement of assessments between raters.^8^ In addition, Tudiver et al. developed the OSCE for interns, reporting excellent construct validity and inter-rater reliability.^9^

Conversely, some studies have reported the OSCE’s inter-rater reliability as problematic. Harasym et al. reported that, in the OSCE concerning medical communication ability, assessed results differ depending upon the strictness or tolerance of raters.^10^ Moreover, Setyonugroho et al. reported an intraclass correlation coefficient (ICC) of 0.45 for inter-rater agreement in a systematic review of the OSCE assessing medical students’ communication skills, indicating a low degree of agreement.^11^ In a study of the reliability of the OSCE for physical and occupational therapists,^12^ Sakurai et al. reported that the ICCs of the two raters were low for some clinical skills tasks, 0.42 for the standing movement assistance task, and 0.34 for the walking assistance task.

Several studies have reported that rater training intervention effectively increases the reliability of OSCE raters. Pell et al. reported that OSCE raters become more accurate when trained, and gender inequality also decreases.^13^ Holmboe et al. suggested that OSCE assessment training involving mini-lectures, small group work, practice assessments using videos, and standardized practice assessments involving interns and simulated patients improve assessment consistency.^14^ Notably, Lin et al. reported that discussions between OSCE raters and roleplay training improve inter-rater reliability.^15^

Frame-of-reference (FOR) training has gained popularity as a method of training for performance assessment.^16^ FOR training is a method of rater training that involves the following procedure: (a) instruction in primary performance indices and behavioral criteria for each index, (b) discussions concerning different levels of behavioral criteria for various indices, (c) hands-on practice assessments using the new assessment criteria created through the discussions, and (d) feedback on assessment accuracy.^17^

Several studies on FOR training have claimed its effectiveness in increasing assessment reliability in performance assessments. Schleicher et al. reported that FOR training for raters improved reliability, accuracy, convergent and discriminant validity, and criterion-related validity in performance evaluation for assessment center exercises. The exercises consisted of presentations, group discussions, and mock interviews using a seven-point behavioral rating scale.^17^ In a study in which psychology department students assessed human behavior in the workplace using a five-point scale, Lievens reported that raters who received FOR training had better inter-rater reliability, rater accuracy, and rating discriminant validity than those who did not receive FOR training.^18^ Hemmer et al. reported that FOR training participants were more accurate in their performance ratings than nonparticipants in which medical students’ clinical skills were rated on a 17-item, 5-point scale.^19^ However, there are no studies concerning FOR training for the raters of OSCE in physical therapy education. Moreover, the effects of FOR training on assessment accuracy have not been clarified.

This study examined the effect of online FOR training on the assessment accuracy of raters of the OSCE for physical therapists.

## Methods

### Participants

The study participants were 36 physical therapists with 5–20 years of experience who worked at nine hospital facilities affiliated with the institution where the study was carried out. None of the participants had experience assessing clinical skills using the OSCE. Their mean amount of clinical experience was 9.4±3.6 years. The participants had no conflicts of interest with the institution where the study was completed.

The study was performed with the approval of the Fujita Health University Research Ethics Review Committee (HM19-462). Participants provided written consent following a full explanation of the purpose and content of the present study in oral and written form. All data were anonymized, such that they could not be traced back to the participants.

### Preparation for the experiment

This study used the OSCE textbook designed for physical and occupational therapists.^20^ It comprises questions, assessment criteria, simulated patient information, and a simulated patient behavior manual. The time for each examination is 5 minutes. The assessment criteria are based on a rubric (Table 1). The 10–15 items comprising each task are assessed on a three-point scale from 0 to 2. In this study, the OSCE task was a manual muscle test (MMT) of hip abduction. MMT is used globally in physical therapy.^21^ Frese et al. reported that MMT of the gluteus medius to abduct the hip has low inter-rater reliability and is a difficult aspect of skill.^22^ Therefore, the gluteus medius MMT to abduct the hip joint was used as the task.

In this study, raters performed assessments after watching videos of the OSCE. The OSCE assessment videos portrayed a character in the role of a student carrying out the MMT task on standardized patients, shot from four angles with fixed video cameras, and displayed simultaneously.

We created the six different scenarios in which the student and simulated patient role acts in the videos of the OSCE. The scenarios were set such that the score of correct answers was distributed and the total score varied. This design was used so that if the correct answers score were biased, the inter-rater agreement would be high (Table 2).

### Experimental procedure

**Figure 1:**
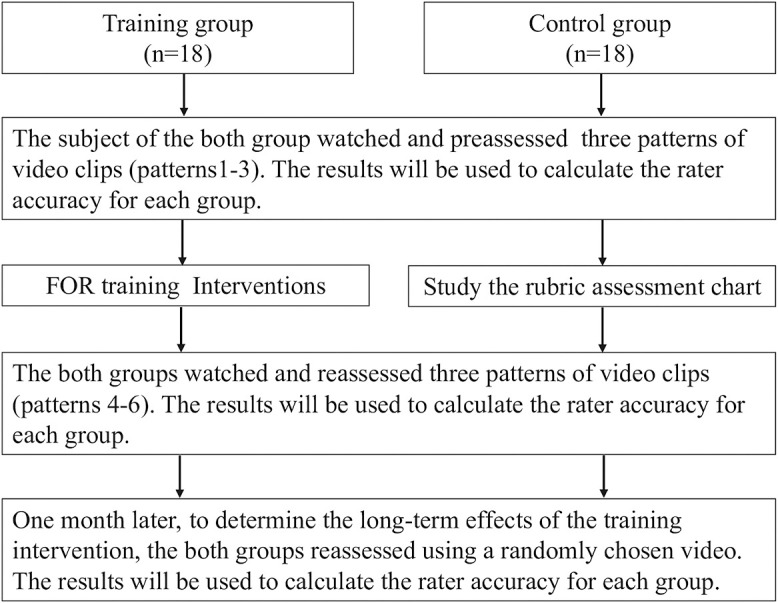
Experimental procedure. The experimental procedure is shown in the flowchart below. The experiment was conducted by dividing the participants into training and control groups.

Participants were randomly separated into two groups: a training group and a control group (Table 3). There was no difference in the number of years of clinical experience between the two groups. Figure 1 shows the experimental procedure. Both groups watched three videos (of patterns 1–3) and performed pre-assessments. The participants performed an OSCE assessment while watching the videos displayed on a computer. Each pattern was played twice. Raters were prohibited from pausing the videos, performing a slow-motion reproduction, or discussion with other participants.

After the two 90-minute sessions training was administered to the training group, both groups watched and assessed three different videos (of patterns 4–6). After 1 month, participants performed a follow-up assessment to determine whether the training intervention’s effect was maintained. For this follow-up assessment, videos with patterns 1, 3, and 4 were selected. These patterns were selected because of the different behavior patterns of the student roles and the different total scores.

### Training methods

Following Schleicher et al., the FOR training conducted in this study consisted of two feedback sessions provided by the facilitator, four group discussions, and rewatching the videos used in the pre-assessment.^17^ The first point in the feedback was regarding the assessment items that had a high variance in scores in the pre-assessment. Afterward, the facilitators were assigned to groups, and group discussions were carried out twice. The duration of each discussion was 90 minutes. The goal of the first group discussion was that the participants construct the assessment criteria, and the second was focused on maintaining and enhancing the assessment criteria. After discussing, the participants rewatched the three patterns shown in the pre-assessment, and the discussion resumed. Following these activities, the participants discussed the scenario and the feedback provided by the facilitator regarding the perspectives of the assessment criteria. Finally, the participants discussed the assessment criteria again.

Although most of the FOR training reported in previous studies had been conducted in person, this study used the online conferencing application, Zoom (Zoom Video Communications Inc., San José, CA, USA) and connected via the Internet to a remote location where the facilities related to this study were located.

The control group received an explanation of the rubric assessment chart and was also asked to complete self-learning to facilitate their understanding of it. Self-learning methods were not provided.

### Statistical analysis

**Figure 2:**
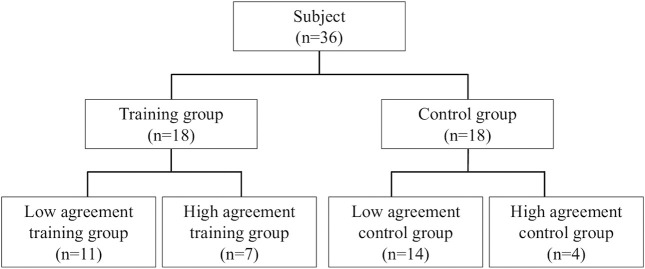
Grouping procedure based on pre-assessment. The participants were divided into training and control groups and, after pre-assessment, both groups were subdivided into two groups, one with low agreement and the other with high agreement.

Based on the assessment results of the training and control groups, raters’ accuracy in the OSCE pre-, post-, and follow-up assessments were determined. As an index of accuracy, the agreement between correct assessments and participants’ assessments of the 15 items of the MMT task in each pattern was calculated using a weighted kappa coefficient. Nicole and Koval reported a value of 0.6≤κ<1 for Cohen’s kappa coefficient, a measure of agreement in small samples, which was substantial to almost perfect. Therefore, the training and control groups were further subdivided into a high agreement group (one with a pre-assessment kappa coefficient of 0.6≤κ) and low agreement group (one with a pre-assessment kappa coefficient of κ<0.6).^23,24^ The training and control groups were compared. Subsequently, we divided the samples into high- and low-agreement groups based on the Cohen, and Nicole and Koval reports. Subsequently, the four groups (low-agreement training group, high-agreement training group, low-agreement control group, and high agreement control group) were compared (Figure 2).

To examine the assessment accuracy, the agreement between the correct scores and the participant’s assessed scores was calculated as the kappa coefficient. A two-way repeated measure analysis of variance was used to analyze the effects of time (pre-, post-, and follow-up assessments) and group (training and control groups). If no interaction was found, a simple main effect was checked. The Bonferroni-correction multiple comparison test was performed when an interaction was not observed in the statistical analysis. IBM SPSS Statistics for Windows, version 26.0 (IBM Corp., Armonk, NY, USA) was used for statistical processing. Statistical significance was set at p<0.05.

## Results

**Figure 3:**
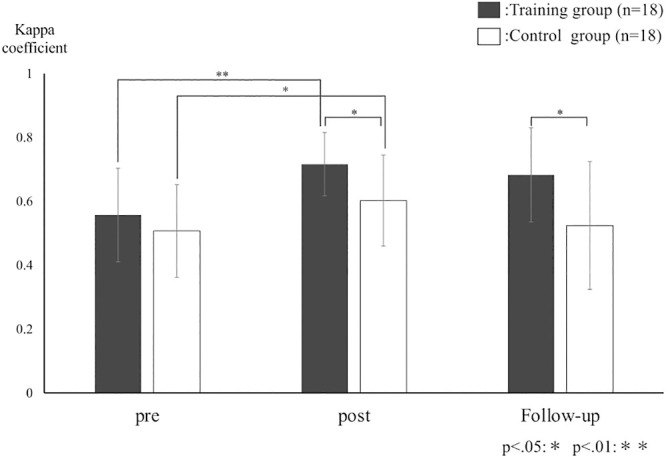
Mean kappa coefficient for the training and control groups. The graph shows the mean kappa coefficient of the training and control groups. The black bar shows the training group, and the white bar shows the control group. The training group showed more improvement in accuracy than did the control group (**).

The kappa coefficient of the training group was 0.56, 0.72, and 0.68 for the pre-, post-, and follow-up assessments, respectively. The kappa coefficient of the control group was 0.51, 0.6, and 0.52 for the pre-, post-, and follow-up assessments, respectively. There was no interaction between the training and control groups [F(2, 68)=1.9, p=0.16], but there were main effects for the assessment of time factors [F(2, 68)=10.4, p<0.05] and the presence or absence of training [F(1, 34)=7.6, p=0.009]. Both groups showed significant differences between pre- and post-assessments. However, there were no significant differences between the post- and follow-up assessments. Even though there were no significant differences between groups at pre-assessment, there were significant differences between groups at the post- and follow-up assessments (Figure 3).

The kappa coefficient of the low-agreement training group was 0.47 for the pre-, 0.72 for the post-, and 0.68 for the follow-up assessments. The kappa coefficient of the low-agreement control group was 0.45 for the pre-, 0.59 for the post-, and 0.47 for the follow-up assessments. The kappa coefficient of the high-agreement training group was 0.7 for the pre-, 0.71 for the post-, and 0.69 for the follow-up assessments. The kappa coefficient of the high-agreement control group was 0.69 for the pre-, 0.64 for the post-, and 0.72 for the follow-up assessments (Table 4).

In the training and control groups with a low agreement for the pre-assessment, significant differences were found in the assessment of time factors [F(2, 46)=17.8, p<0.05], presence or absence of training [F(1, 23)=6.2, p=0.02], and interaction [F(2, 46)=4.6, p=0.02]. According to the Bonferroni-correction multiple comparison test, significant differences were found between the pre-assessment and post-assessment (p<0.05) and between the pre-assessment and follow-up assessment (p=0.001) for the training group. For the control group, significant differences were found between the pre-assessment and post-assessment (p=0.002) and between the post-assessment and follow-up assessment (p=0.05). Moreover, a significant difference was found between the two groups’ post-assessment (p=0.04) and follow-up assessment (p=0.01).

**Figure 4:**
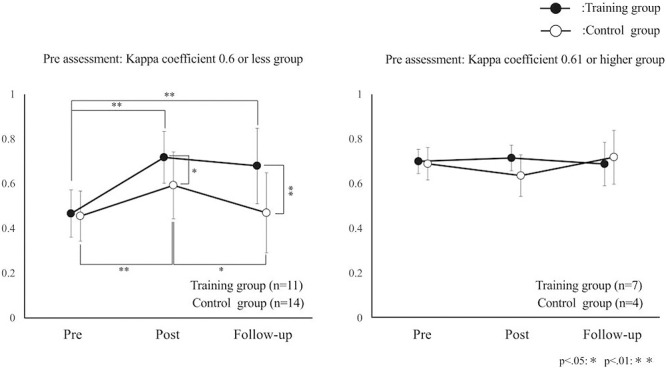
Mean kappa coefficient for each group. The graph shows the mean kappa coefficient of the four groups. Black plots show the training group, and white plots show the control group. The left graph shows the participants with low agreement, and the right graph shows the participants with high agreement. The participants with low agreement showed an improvement in accuracy in the training group (**). The control group showed an improvement in accuracy temporarily (**). The participants with high agreement do not show a change in accuracy in either group.

In the training and control groups with a high agreement for the pre-assessment, no significant difference was found for the assessment of time factors [F(2, 18)=0.31, p=0.73], presence or absence of training [F(1, 9)=0.25, p=0.63], or interaction [F(2, 18)=1.2, p=0.33] (Figure 4).

## Discussion

In the present study, investigating the effects of online FOR training on raters’ assessment accuracy of the OSCE in physical therapy education showed that the training group accuracy of the clinical assessment improved more than the control group, and that assessment accuracy improved in low-agreement training and control groups at the pre-assessment level. Additionally, assessment accuracy for the low-agreement training group was higher than the low-agreement control group at the post- and follow-up assessments.

The assessment criteria of the OSCE used in the present study are based on a rubric. The rubric assessment incorporates a performance task perspective, consists of clear assessment criteria and scales, and can reduce inequality in raters’ assessments. Kilgour et al. reported that rubrics encourage high assessment accuracy.^25^ However, Bergin et al. confirmed that using a rubric effectively increases assessment accuracy but observed that clustering occurs when groups with different degrees of assessment accuracy take shape.^26^ Furthermore, regarding FOR training, Athey and McIntyre reported that assessment criteria are reconstituted based on discussion and feedback, thus increasing assessment accuracy.^27^ Based on this previous study, the online FOR training group in this study improved and maintained its accuracy better than the control group because of the sharing of correct answers to test questions and assessment criteria among raters through group discussions and feedback on the rating videos. Gorman and Rentsch reported that raters who received two sessions of 45-minute FOR training could maintain their assessment criteria for judging performance and maintain assessment accuracy 2 weeks after the training.^28^ This study’s results were consistent with and supportive of those of Gorman and Rentsch.

However, online FOR training was not found effective for the training and control groups that already had high accuracy at the pre-assessment. This observation is likely the result of two factors. The first factor is the degree of difficulty of the FOR training. Stockdale and Williams reported that the same educational intervention in a group of undergraduate educational psychology students divided into high, average, and low test performance groups resulted in a significant improvement in the performance of the low-performing group but a decline in the performance of the high-performing group.^29^ Navarro et al. reported results in mathematics education for preschoolers in which similar educational interventions for high- and low-achieving students in the early years improved the performance of high-achieving preschoolers. However, the rate of improvement was lower than that of low-achieving preschoolers.^30^ These studies show that accuracy did not improve as the degree of difficulty of the online FOR training program was not appropriate for raters already capable of accurate assessment at the pre-assessment. The second factor is the assessment using videos. Yeates et al. reported that the successful use of video-based assessment relies on balancing the need to ensure station-specific information adequacy.^31^ In the OSCE for physical therapists, assessment criteria often include the power, direction, and timing of force applied to patients’ bodies as technical elements. These assessment criteria are unique as they are difficult for raters to confirm based on visual information alone and are highly subjective. The MMT task used in the present study includes multiple factors such as means of keeping the patient in the correct posture and applying resistance in the correct direction and control of compensatory movements, as mentioned in assessment items 6, 8, 10, and 11 in Table 1. In this study, four-angle videos were assessed, but it is possible that the information necessary for assessing these factors was insufficient. Moreover, in the present study, not all groups had a kappa coefficient of 0.81 or above, a degree of agreement referred to as “almost perfect.” Based on these results, the video-based assessments, which are difficult to assess through visual information alone, seem to influence the effects of the online FOR training in the present study, regardless of the accuracy of the pre-assessment. To increase the effectiveness of online FOR training in the future, programs must be devised based on raters’ abilities. In addition, videos that are difficult to judge should be re-edited to make them easier to assess.

In the present study, FOR training was carried out online. Online lectures and training are said to have the merits of simplicity and low cost, as do methods such as feedback and group discussion.

The limitations of this study lie in the fact that the OSCE administered was limited to the investigation of a single task, MMT, and that it was not possible to discuss the numerous technical tasks of physical therapists. As previously described, as physical therapy techniques have special characteristics, in the future, it will be necessary to optimize the assessment criteria for other techniques while investigating the effects of FOR training.

## Conclusion

In the present study, it became evident that online FOR training improved the assessment accuracy of the OSCE raters in physical therapy education who had low accuracy before training, and this improvement was maintained. Furthermore, online FOR training was not found effective for raters with high assessment accuracy before training; the effect of online FOR training was limited to a kappa coefficient of below 0.8. These results suggest that to further improve the effectiveness of online FOR training in the future, it will be necessary to devise a program suited to raters’ abilities and re-edit difficult-to-judge videos to make them easier to assess. It will also be necessary to verify the effectiveness of online FOR training in different OSCE tasks.

## Figures and Tables

**Table1:** Objective Structured Clinical Examination Scoring Rubric Table for Manual Muscle Testing*

Skill Scoring Item	Task(s): The Examinee...	2 points	1 point	0 points
1	Can place the patient in the appropriate measurement position (supine or lateral).	Can complete both tasks.	Can do only one task or the other.	Cannot do either task.

2	Can verbally confirm the left–right difference to the patient and take measurements in the proper order.	Can complete all the tasks.	Checks the patient for left–right differences in muscle strength, but measures from the side with the weak muscle strength.	Does not check the patient for left–right differences in muscle strength.

3	Can check the patient’s active lower extremity movement, pain, and muscle tone both sides.	Can complete all the tasks.	Can perform the tasks on only one side.	Cannot do the tasks on either side of the measurement limb.

4	Can check the patient’s passive lower extremity movement, pain, and muscle tone both sides.	Can complete all the tasks.	Can perform the tasks on only one side.	Cannot do the tasks on either side of the measurement limb.

5	Can explain exercise, measurement methods, and compensatory movements to the patient with demonstrations.	Can complete all the tasks.	Cannot do one item, or it can only be explained orally.	Cannot do more than two tasks.

6	Can immobilize the patient’s pelvis so that no compensatory movements appear in the Stage 3 testing both sides.	Can complete all the tasks.	Can perform the tasks on only one side.	Cannot do the task on either side of the measurement limb.

7	Can perform the Stage 3 testing both sides and can remeasure if compensatory movements occur in the patient.	Can complete all the tasks.	Can do the tasks on both sides but not remeasure if compensatory movements occur or can perform the testing on only one side.	Cannot do the task on either side.

8	Can immobilize the patient’s pelvis so that no compensatory movements appear in the Stage 4 and Stage 5 testing both sides.	Can complete all the tasks.	Can perform the tasks on only one side.	Cannot do the task on either side of the measurement limb.

9	Can apply resistance to the lateral aspect of the patient’s knee joint in Stage 4 and Stage 5 testing.	Can complete all the tasks.	Can perform the tasks on only one side.	Cannot do the tasks on either side of the measurement limb.

10	Can apply resistance to the vertical direction in Stage 4 and Stage 5 testing.	Can complete all the tasks.	Can perform the tasks on only one side.	Cannot do the tasks on either side of the measurement limb.

11	Can apply resistance to the patient in Stage 4 and Stage 5 testing, varying from weak resistance to maximum resistance.	Can complete all the tasks.	Can perform the tasks on only one side.	Cannot do the tasks on either side of the measurement limb.

12	Can apply resistance to the patient for about 2–3 seconds in Stage 4 and Stage 5 testing.	Can complete all the tasks.	Can perform the tasks on only one side.	Cannot do the tasks on either side of the measurement limb.

13	Can verbally instruct the patient appropriately to get to maximum muscle strength during the measurement.	Can complete all the tasks.	Can speak to the patient, but it is not an appropriate voice for maximal muscle strength.	Cannot speak to the patient.

14	Can appropriately determine the patient’s muscle strength level from the measurement results.	Can complete all the tasks.	Can judge only one side accurately.	Cannot judge either side accurately.

15	Can explain the results to the patient clearly.	Can complete all the tasks.	Can inform the patient of the measurement results, but the explanation is unclear.	Incorrectly informs the patient of measurement results.

* Manual muscle testing was performed on the gluteus medius (hip abduction).

**Table2:** Details of the Six Video Scenarios (Patterns)*

Item	Tasks: The Examinee...	Score					
		Pattern 1	Pattern 2	Pattern 3	Pattern 4	Pattern 5	Pattern 6
1	Can place the patient in the appropriate measurement position (supine or lateral).	2	2	2	2	2	2

2	Can verbally confirm the left–right difference to the patient and take measurements in the proper order.	2	0	2	1	0	2

3	Can check the patient’s active lower extremity movement, pain, and muscle tone both sides.	0	1	0	0	2	0

4	Can check the patient’s passive lower extremity movement, pain, and muscle tone both sides.	0	2	0	1	0	2

5	Can explain exercise, measurement methods, and compensatory movements to the patient with demonstrations.	2	0	0	1	0	2

6	Can immobilize the patient’s pelvis so that no compensatory movements appear in the Stage 3 testing both sides.	2	0	0	1	0	2

7	Can perform the Stage 3 testing on both sides and can remeasure if compensatory movements occur in the patient.	2	0	0	1	0	2

8	Can immobilize the patient’s pelvis so that no compensatory movements appear in the Stage 4 and Stage 5 testing both sides.	2	0	0	1	0	2

9	Can apply resistance to the lateral aspect of the patient’s knee joint in Stage 4 and Stage 5 testing.	2	0	0	0	1	2

10	Can apply resistance to the vertical direction in Stage 4 and Stage 5 testing.	2	1	2	2	0	2

11	Can apply resistance to the patient in Stage 4 and Stage 5 testing, varying from weak resistance to maximum resistance.	0	1	2	0	1	2

12	Can apply resistance to the patient for about 2–3 seconds in Stage 4 and Stage 5 testing.	0	1	1	0	1	2

13	Can verbally instruct to the patient appropriately to get to maximum muscle strength during the measurement.	2	1	1	0	1	2

14	Can appropriately determine the patient’s muscle strength level from the measurement results.	2	1	0	2	1	2

15	Can explain the results to the patient clearly.	2	1	1	2	1	2

	Total score	22	11	11	14	10	28

* The patterns of behavior were set up such that the students’ simulated clinical skills varied.

**Table3:** Number of participants in each group and years of experience

	n	Number of years of clinical experience
Training Group	18	10.0±4.1
Control Group	18	8.8±3.1

**Table4:** Average kappa coefficient of the four groups

Group	kappa coefficient		
	Pre-Assessment	Post-Assessment	Follow-up Assessment
Low-agreement training group	0.47±0.1	0.72±0.12	0.68±0.17
Low-agreement control group	0.45±0.11	0.59±0.15	0.47±0.18
High-agreement training group	0.70±0.05	0.71±0.06	0.69±0.1
High-agreement control group	0.69±0.07	0.64±0.09	0.72±0.12
